# Dequalinium Chloride Effectively Disrupts Bacterial Vaginosis (BV) *Gardnerella* spp. Biofilms

**DOI:** 10.3390/pathogens10030261

**Published:** 2021-02-25

**Authors:** Carlos Gaspar, Joana Rolo, Nuno Cerca, Rita Palmeira-de-Oliveira, José Martinez-de-Oliveira, Ana Palmeira-de-Oliveira

**Affiliations:** 1 CICS-UBI—Health Sciences Research Center, University of Beira Interior, 6200-506 Covilhã, Portugal; cgaspar@fcsaude.ubi.pt (C.G.); joanarolo@fcsaude.ubi.pt (J.R.); rpo@fcsaude.ubi.pt (R.P.-d.-O.); jmo@fcsaude.ubi.pt (J.M.-d.-O.); 2 Faculty of Health Sciences, University of Beira Interior, 6200-506 Covilhã, Portugal; 3 Labfit-HPRD—Health Products Research and Development Lda, 6200-506 Covilhã, Portugal; 4 Centre of Biological Engineering (CEB), Laboratory of Research in Biofilms Rosário Oliveira (LIBRO), University of Minho, Campus de Gualtar, 4710-057 Braga, Portugal; nunocerca@ceb.uminho.pt; 5 CNC—Center for Neuroscience and Cell Biology, University of Coimbra, Rua Larga, 3004-504 Coimbra, Portugal

**Keywords:** bacterial vaginosis, dequalinium chloride, biofilm, treatment, recurrence prevention

## Abstract

Bacterial vaginosis (BV) is the most frequent vaginal infection worldwide. It is caused by the overgrowth of anaerobic vaginal pathogens such as *Gardnerella* spp. BV has been associated with the occurrence of dense multispecies biofilms on the vaginal mucosa. Treatment of biofilm-associated infections such as BV is challenging. In this study, we have tested the role of a quaternary ammonium compound, dequalinium chloride (DQC), in the eradication of *Gardnerella* spp. biofilms. The effects of the test substance on the biomass and the metabolic activity of the biofilm of *Gardnerella* spp. were assessed in vitro using a microtiter plate assay. In addition, the effect of DQC on the *Gardnerella* spp. biofilm was further assessed by using scanning electron microscopy and confocal laser scanning microscopy. The results showed that DQC was particularly effective in the destruction of BV-associated *Gardnerella* spp. biotypes, impacting both their biomass and metabolic activity. In addition, the disruption of biofilm architecture was evident and was probably caused by multiple mechanisms of action. We conclude that DQC is an antibiofilm agent and is able to efficiently destroy *Gardnerella* spp. BV-associated biofilms. Therefore, it is a valid option for BV therapy and has the potential to prevent BV recurrences.

## 1. Introduction

Biofilms are complex arrays of microorganism cells, assembled by superficial proteins and exopolysaccharides [1]. Due to the structure of biofilms, the efficacy of antimicrobial compounds against biofilms is generally reduced. A wide array of microbial pathogens has been known to form biofilms on mucosa [2] and abiotic structures [3], preventing effective treatment of infections. Consequently, there has been increasing interest in research on new compounds with antibiofilm activity [4].

*Gardnerella* spp. and other anaerobes are vaginal pathogens that have been associated with the onset of bacterial vaginosis (BV) [5,6]. Recently, it has been suggested that several species of the genus *Gardnerella* can be involved in this vaginal infection [5]. BV is the most common vaginal infection, impacting women’s sexual health and quality of life worldwide. It is characterized by an exaggerated vaginal discharge, often accompanied by a characteristic strong odor [7]. Previous studies have established that *Gardnerella* spp. isolated from either BV or non-BV/asymptomatic cases are phenotypically different [8]. These have been extensively characterized before; specifically, BV-associated *Gardnerella* spp. have been shown to produce biofilm [8] and have an increased expression of other virulence factors, leading, for example, to higher cytotoxicity against epithelial cells [5]. The treatment of BV is still challenging, particularly because of the need to eradicate biofilms [6]. Currently, the most commonly prescribed drugs are metronidazole and clindamycin [9]. However, difficulties occur when using these drugs, namely the emergence of resistance to treatment [10] and the inability to eradicate vaginal biofilms [10], which often lead to treatment failure and BV recurrences. This resistance to treatment can be further complicated by the complex vaginal microbiota, which contains multiple species. Treatment with antibiotics causes a shift in the composition of the vaginal microbiome, leading to the emergence of antibiotic-resistant pathogens [11].

Dequalinium chloride (DQC) is a quaternary ammonium salt with antimicrobial activity that has been mostly used as an antiseptic in the clinical context [12]. DQC also has some anti-inflammatory properties [13]. It has been previously demonstrated that the compound is active against a wide range of vaginal pathogens, including planktonic cells of *Gardnerella* spp. [13,14]. In addition, because DQC has been described to show multiple modes of action against bacteria (disruption of cell permeability and enzymatic inactivation) [13], the risk for emergence of resistance is considered lower. Likewise, the dominance of the microbiome by resistant bacteria should be a rare event. Furthermore, toxicology assessment studies have revealed that systemic absorption of DQC is negligible [15], supporting its safety in clinical use, even during pregnancy.

Recently, DQC has been demonstrated as a successful treatment of various clinical cases of BV [16]. This study aims to add to the knowledge on the efficacy of DQC in the treatment of BV, with a particular focus on its role in the eradication of *Gardnerella* spp. biofilms.

## 2. Methods

### 2.1. Bacterial Strains and Growth Conditions

Five *Gardnerella* spp. strains previously obtained in a different study from several women’s vaginal tracts were included [8]. Due to their virulent characteristics, the strains were previously classified as BV (*Gardnerella vaginalis*, UM137; *Gardnerella* spp. UM241) and non-BV (*Gardnerella vaginalis*, UM085; *Gardnerella* spp. UM131; *Gardnerella* spp. UM246) strains [8,17]. The strains were subcultured twice in Colombia Blood Agar (bioMérieux, Marcy L’Étoile, France) before all experiments were conducted (total incubation time of 48 h at 37 °C with 10% CO_2_).

### 2.2. Test Substances

Dequalinium chloride (DQC) was prepared by dispersing a tablet of the compound as it is commercialized (Fluomizin, Medinova, Zürich, Switzerland) in ultrapure water at 1024 µg/mL. The concentration of the compound used for the experiments was based on the minimum inhibitory concentrations previously determined (2–512 µg/mL) [13,14]. Clindamycin (IV solution, Labesfal, Santiago de Besteiros, Portugal) was diluted with ultrapure water at 150 mg/mL.

### 2.3. Culture Conditions for Gardnerella *spp.* Biofilm Formation

Two different culture media were used: (1) Supplemented Brain Heart Infusion (BHI) medium: BHI medium (VWR, Avantor, PA, USA), 2% (*w*/*v*) gelatin (Sigma-Aldrich, Hamburg, Germany), 1% (*w*/*v*) yeast extract (VWR, Avantor, PA, USA), and 0.1% (*w*/*v*) soluble starch (Merck, Gernsheim, Germany); (2) New York City III (NYCIII) medium: 0.4% (*w*/*v*) HEPES buffer (Merck, Gernsheim, Germany), 1.5% (*w*/*v*) proteose peptone (VWR, Avantor, PA, USA), 0.5% (*w*/*v*) sodium chloride (VWR, Avantor, PA, USA), 0.5% (*w*/*v*) glucose (VWR, Avantor, PA, USA), 2.5% (*w*/*v*) yeast extract (VWR, Avantor, PA, USA), and 10% (*v*/*v*) fetal bovine serum (FBS) (Sigma-Aldrich, Hamburg, Germany). The incubation conditions used were as follows: 24 h, 10% CO_2_, and 37 °C (Binder GmbH, Tuttlingen, Germany).

### 2.4. Evaluation of Gardnerella *spp.* Biofilm Metabolic Activity (MTT) and Biomass (CV)

A pre-inoculum was performed by growing the strains in NYCIII (incubation for 24 h at 37 ℃ with 10% CO_2_). The cellular concentration of the pre-inoculum was adjusted to 1 × 10^8^ CFU/mL, and the pre-inoculum deposited in flat-bottomed untreated plastic 96-well plates (in duplicate—two wells per strain/condition). The incubation conditions of the plates to allow biofilm formation were 48 h, 10% CO_2_, and 37 ℃ (Binder GmbH, Germany). Following this incubation, the biofilms were washed three times with sterile PBS (phosphate buffered saline) 1× (Sigma-Aldrich, Germany). Afterward, the test substance (dequalinium chloride or clindamycin) was applied. Seven symmetric concentrations (half-log intervals: 0.26, 0.81, 2.57, 8.11, 25.64, 81.01, and 256 µg/mL) were tested in triplicate including a positive control (that consisted in culture medium only). The incubation proceeded for 24 h at 37 °C with 10% CO_2_. Afterward, the biofilms were washed three times with sterile PBS 1× in order to remove traces of the test substances. The quantification of the biofilm biomass was performed by fixing the biofilms with methanol (VWR, Avantor, USA) for 30 min at room temperature and staining the biomass with crystal violet (0.01%, *w*/*v*) for 30 min at room temperature [18]. Absorbance was read at 630 nm (BioRad, Hercules, CA, USA). The metabolic activity of the biofilm was determined by incubating the biofilm with culture media supplemented with 1 mg/mL MTT (Sigma-Aldrich, Germany) for 4 h in 10% CO_2_ at 37 °C. The resulting formazan crystals were resuspended with DMSO (dimethyl sulfoxide) (Sigma-Aldrich, Germany) [18]. Absorbance was read at 490 nm (BioRad, USA). The positive control was normalized at 100% biomass/cell viability, and the absorbance values obtained in each assay were normalized considering the absorbance values obtained for the positive control.

### 2.5. Visualization of the Effect of Dequalinium Chloride Against Formed Gardnerella *spp.* Biofilms

Biofilms were formed as specified above in a glass coverslip. After the treatment with the test substance on a glass coverslip, the biofilm was fixed overnight with 3% (*v*/*v*) glutaraldehyde (VWR, Avantor, USA) solution in PBS at 4 ℃. Post-fixation of the biofilm was done by a 2% (*v*/*v*) osmium tetroxide solution (Sigma-Aldrich, Germany) for 1 h at room temperature. After each fixation, bacterial cells were washed twice with PBS and dehydrated with ethanol (VWR, Avantor, USA) 25%, 50%, 75%, and 100% for 15 min each. In addition, the samples were dried overnight using a freeze-drying process (BioGene lyophilizer freeze dryer, BioGene Biotechnologies, Ghaziabad, India). Each sample was mounted on double-sided tape glued to an aluminum supporter, and the samples were gold-covered using an Ion Coater (Quorum model Q 150R ES, Quorum Technologies, United Kingdom). Finally, the preparations were observed using a VP SEM Hitachi S-3400N (Hitachi Instruments, Inc., San Jose, CA, USA) scanning electron microscope (SEM).

### 2.6. Study of the Ability of DQC to Penetrate Biofilms of Gardnerella *spp.*

To study the ability of DQC to penetrate *Gardnerella* spp. biofilms, the compound was stained with fluorescein isothiocyanate (FITC). Biofilms were formed as above. After the incubation period, the biofilms were washed and treated with the test substance stained with FITC or with the substance alone. Staining with the DQC–FITC complex was performed by adding a solution of 1 mg/mL of DQC to a solution of 2 mg/mL of FITC in ultrapure water. The mixture was allowed to mix at pH 3 and was further neutralized at pH 6. The DQC–FITC complex formed was found to be insoluble and therefore was lyophilized overnight using a freeze-drying process (BioGene lyophilizer freeze dryer, BioGene Biotechnologies, India). The complex was then resuspended at 256 µg/mL in NYCIII culture media with 1% DMSO (Sigma-Aldrich, Germany), and the resulting mixture was used to treat preformed biofilms. The biofilms were treated and washed as described above. After the washes, 10 µg/mL propidium iodide (Sigma-Aldrich, Germany) and 5 mM SYTO40 (Sigma-Aldrich, Germany) were added sequentially. Images were obtained in a confocal laser scanning microscope (Zeiss LSM 710, Aalen, Germany). A control was performed by killing the biofilm cells with 70% ethanol (VWR, Avantor, USA) for 30 min.

### 2.7. Analysis of Results

The results presented represent at least two independent experiments and are expressed as the mean and the respective standard deviation. The distribution of the concentrations obtained for 50% of the destruction of the biofilm biomass (Biomass EC_50_), as well as the concentrations obtained for 50% of the metabolic activity (Metabolic EC_50_), for the strains from each clinical group (BV vs. non-BV) were analyzed for their statistical significance using an unpaired Student’s t-test. A paired Student’s *t*-test was used to analyze each pair of results (clindamycin vs. DQC) for each strain at each concentration tested. Statistically significant results were considered when *p* < 0.05.

## 3. Results and Discussion

### 3.1. Comparison of the Gardnerella *spp.* Ability to form Biofilms Using Two Culture Media

**Figure 1:**
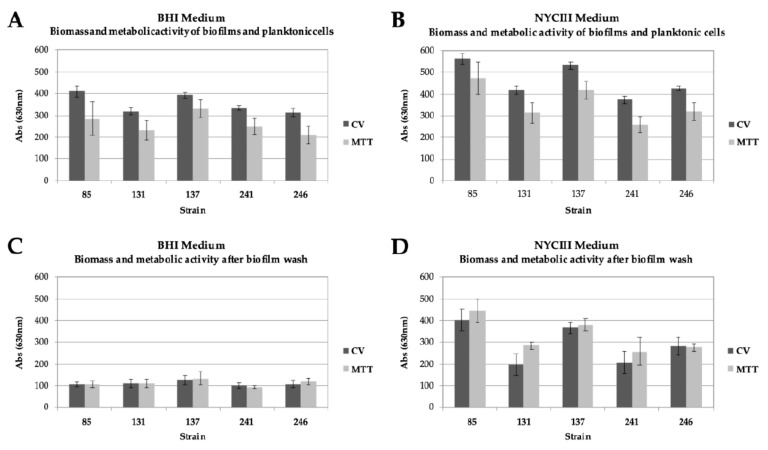
Comparison of the optical density of the biofilms of *Gardnerella* spp. strains using two different culture conditions. (**A**). Absorbance of biofilm and planktonic cells on BHI culture medium. (**B**). Absorbance of biofilm and planktonic cells on NYCIII culture medium. (**C**). Absorbance of biofilm and planktonic cells on BHI culture medium, after the washes with PBS 1x. (**D**). Absorbance of biofilm and planktonic cells on NYCIII culture medium, after the washes with PBS 1×. CV: crystal violet (indicative of biofilm biomass; absorbance was read at 630 nm); MTT: conversion of the tetrazolium dye MTT to formazan crystals (indicative of metabolic activity; absorbance was read at 490 nm). The results shown represent the mean of at least two different independent assays. The respective standard deviation is also shown.

Culture conditions strongly influence in vitro formation *of Gardnerella* spp. biofilms [19]. Therefore, two different culture conditions were tested in order to choose the conditions that provided the most robust biofilm in order to assess the effect of DQC on its structure. Our results revealed that both culture media can sustain *Gardnerella* spp. biofilm growth (Figure 1). In addition, the biofilm obtained using NYCIII medium exhibited an absorbance value that was 4 times higher than the one obtained using BHI medium. These results demonstrate that NYCIII medium provides a higher number of cells in biofilms, which is indicative of a potentially higher *Gardnerella* spp. growth rate and, consequently, a more consistent biofilm. In fact, the in vitro capacity of *Gardnerella* spp. isolates to produce biofilms is variable and highly dependent on culture media, as previously described [8]. Therefore, for this study, we selected the NYCIII as the culture medium to produce biofilms to obtain the most robust structure and thus the worst-case scenario for testing the antibiofilm effect of DQC.

### 3.2. Comparison of Antibiofilm Activity of DQC and Clindamycin Against Gardnerella *spp.*

**Figure 2:**
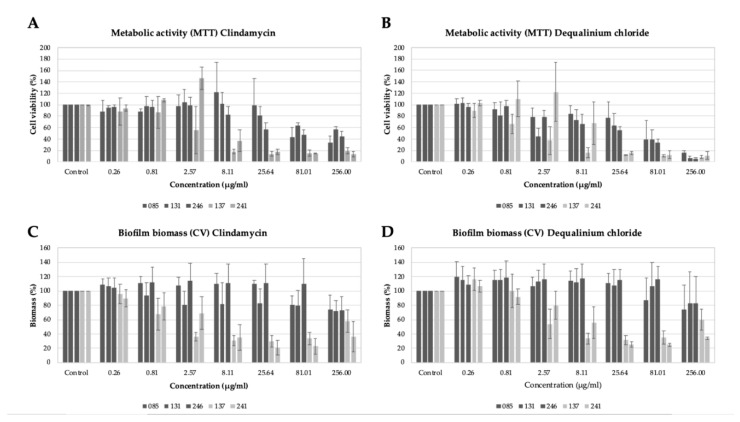
Effect of test substance (dequalinium chloride) and clindamycin on BV and non-BV *Gardnerella* spp. biofilms. (**A**). Metabolic activity (%) of *Gardnerella* spp. biofilms after treatment with clindamycin. (**B**). Metabolic activity (%) of *Gardnerella* spp. biofilms after treatment with dequalinium chloride. (**C**). Biofilm biomass (%) of *Gardnerella* spp. biofilms after treatment with clindamycin. (**D**). Biofilm biomass (%) of *Gardnerella* spp. biofilms after treatment with dequalinium chloride. CV: crystal violet (indicative of biofilm biomass; absorbance was read at 630 nm); MTT: conversion of 3-(4,5-dimethylthiazol-2-yl)-2,5-diphenyltetrazolium bromide to formazan crystals (indicative of metabolic activity; absorbance was read at 490 nm). Dark gray = non-BV strains; light gray = BV strains. The results shown represent the mean of at least two different independent assays. The respective standard deviation is also shown.

The results regarding the effect of DQC on *Gardnerella* spp. biofilm revealed that the substance is particularly effective against the biofilm of BV-associated *Gardnerella* spp. strains (Figure 2) and is able to reduce the metabolism and/or the biomass of these strains by at least 50% (efficient concentration, EC50) at a concentration of 8.11 µg/mL (Table 1) and by 80% at a concentration of 25.64 µg/mL. The results were similar to the ones presented by clindamycin: the EC50 of clindamycin was also 8.11 µg/mL. Statistical analysis considering the entire dataset revealed that the effect of DQC in the biofilm biomass reduction was stronger (*p* < 0.05) than that of clindamycin. Clindamycin had a stronger effect on the reduction of metabolic activity (*p* < 0.05).

The non-BV *Gardnerella* spp. strains showed higher biomass and metabolic EC50; nonetheless, DQC was still effective against these, particularly in the reduction of metabolic activity (Table 1). The differences regarding the Biomass EC50 and Metabolic EC50 between BV and non-BV strains were statistically significant (*p* < 0.05). These differences may be related to the strains’ genotypes.

The prevalence of some *Gardnerella* spp. genotypes in BV clinical cases has been previously reported, along with differences in virulence potential. Specifically, it was previously shown that BV-associated *Gardnerella* spp. have (i) a higher cytotoxicity, (ii) a greater ability to adhere to HeLa cells, and (iii) a greater ability to displace pre-adhered *Lactobacillus crispatus*. Since the effects of the compounds were different depending on the phenotype and/or disease status of the patients from whom the strains were collected, those differences at the strain level might be related to the enhanced virulence capacity of the strains, which could explain their different responses to the compounds [8].

While clindamycin acts by inhibiting protein synthesis [20], quaternary ammonium salts, such as DQC, have been described to perturb microorganisms’ cytoplasmatic membrane, although multiple mechanisms of action have been described and are expected for this molecule (loss of enzymatic activity, for instance) [12]. At lower concentrations (<8.11 µg/mL), metabolic activity was increased, especially in the assays with DQC but also for some strains exposed to clindamycin. The overall increased metabolic activity at low concentrations of the compounds might be a stress-related mechanism employed by the bacteria.

The isolation of clindamycin-resistant vaginal microorganisms has already been reported [21]; therefore, alternative treatments are required. Our results reveal that clindamycin and DQC have the same ability to eradicate *Gardnerella* spp. biofilms in vitro. These results were expected, since the activity of clindamycin and DQC against BV-associated *Gardnerella* spp. planktonic cells has been reported to be fairly similar (128–256 µg/mL) [8,14]. Therefore, besides its reported activity against planktonic *Gardnerella* cells, DQC can also impair *Gardnerella* spp. biofilms with the added advantage of a low emergence of resistance being expected due to its multiple modes of action [12]. Furthermore, the range of concentrations that we found to be effective against BV-associated *Gardnerella* spp. biofilms (25.64 µg/mL) is lower than the concentration of the drug that is usually prescribed (10 mg/day) as a vaginal pill [13]. Therefore, our results corroborate that treatment with DQC can destroy *Gardnerella* spp. biofilms in vivo.

### 3.3. DQC Interferes with Gardnerella *spp.* Biofilms by a Nonspecific Mechanism

The effect on the biofilm of *Gardnerella* spp. was further visualized using SEM by exposing one BV (241) and one non-BV strain (131) to the maximum concentration tested (256 µg/mL) and to the concentration able to reduce 50% of the biofilm biomass of strain 241 (EC50, 8.11 µg/mL, Table 1). These two strains were chosen as representatives of the strain collection since they presented a similar biofilm-forming capacity (Figure 1).

**Figure 3:**
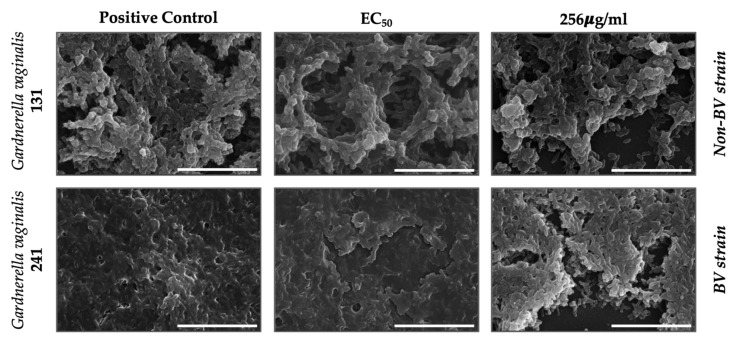
Visualization of the effect of test substance (dequalinium chloride) on the biomass of BV and non-BV *Gardnerella* spp. biofilms, using scanning electron microscopy (SEM). Positive control: culture media only. The scale indicates 10 µm.

The results showed a visible reduction in biofilm architecture (Figure 3). There is a clear loss of cells, evidenced by the large portions of biofilm that detach in comparison with the positive control. The detachment causes the formation of holes in the structure of biofilms. These results are also evidenced by the loss of biofilm biomass (Figure 2). This destruction occurred in both BV- and non-BV-associated biotypes (Figure 2). Since the effect of DQC on planktonic cells is already evident [14], by also destroying biofilm architecture, the test substance is a promising alternative against BV biofilm treatment.

**Figure 4:**
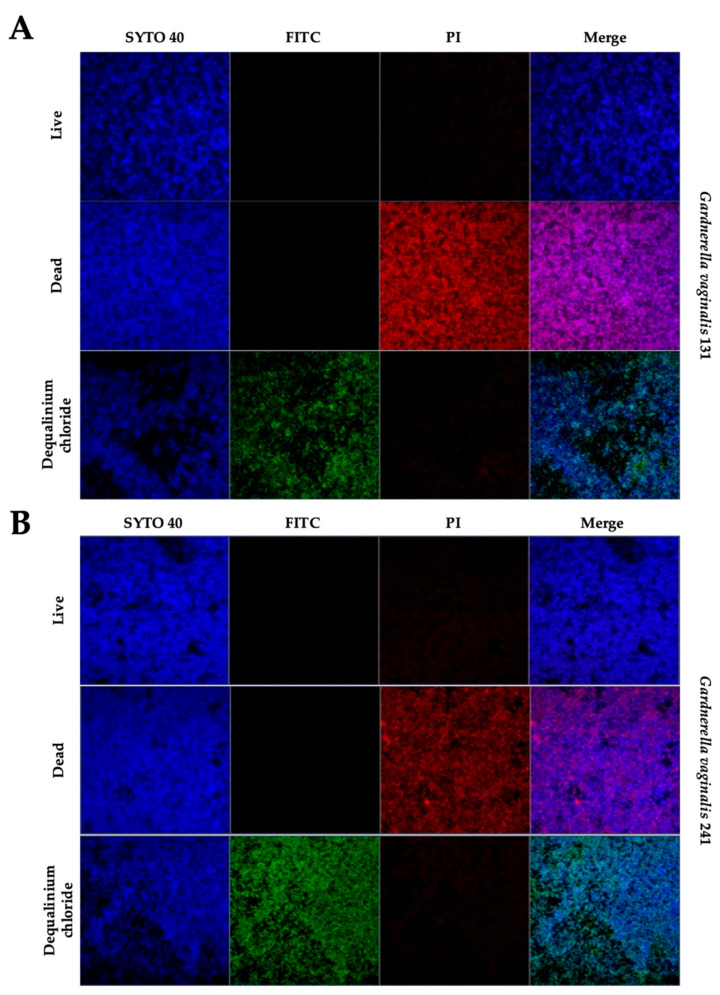
Visualization of the test substance (dequalinium chloride, 256 µg/mL) and interaction with *Gardnerella* spp. biofilm of non-BV strain 131 (**A**) and BV strain 241 (**B**). Green: dequalinium chloride (FITC), blue: nucleic acids (Syto40); red: dead cells (propidium iodide, PI). Live: positive control, culture media was used to form biofilms. Dead: 70% ethanol was used to kill formed biofilms. Dequalinium chloride: treated biofilms with the dequalinium chloride–FITC complex. Magnification: 40×.

The ability of the test substance to penetrate *Gardnerella* spp. biofilms was elucidated by staining DQC with a fluorescent compound (FITC) and also by using a fluorophore indicative of a primary lesion of the membrane (propidium iodide). Nucleic acids were stained with Syto40. The concentration of the test substance stained with FITC used in this test was the maximum concentration tested (256 µg/mL). Confocal microscopy further demonstrated the three-dimensional destruction of the biofilm after DQC treatment (Figure 4 and Supplementary Materials Figure S1). The results show that the DQC–FITC complex is possibly located primarily at the top of the biofilm layer (Figure 4). This result was expected, since the DQC–FITC complex has a greater molecular weight than the test substance alone. FITC is widely used to attach a fluorescent label using the amine group. Its structure attached to the DQC structure is expected to be complex; furthermore, the active groups of the DQC molecule, responsible for its bioactivity, are not probably entirely available. In addition, using this methodology, the investigation of the mechanism of action is hampered by the complex architecture of the biofilm. To overcome this limitation, we performed an additional experiment, in which the biofilm was stained after treatment with DQC (Supplementary Materials Figure S2). By comparing the results with differential staining using different fluorophores, we found that the great majority of the biofilm cells did not incorporate propidium iodide, evidencing that the mechanism of action in the destruction of biofilms is not probably related to the primary lesion of the cellular membrane.

Previous studies revealed that DQC affects planktonic cells by diffusion through the cell wall and binding itself to the cytoplasmatic membrane [22]. Other mechanisms of action include denaturation of proteins [23]. Therefore, lysis of the cytoplasmatic membrane might occur, but probably not at the concentration used in our study. We hypothesize that at this concentration DQC binds to the cytoplasmatic membrane and probably diffuses to the interior of the cells, causing their death and detachment from the biofilm structure. Nonetheless, using confocal microscopy, the results obtained with SEM are corroborated since three-dimensional impairment of the biofilm was observed.

## 4. Conclusions

*Gardnerella* spp. strains are able to form biofilm on vaginal tissue, corresponding to an important mechanism of virulence. Using in vitro methods, we tested the action of DQC on *Gardnerella* spp. biofilms. Our results revealed that DQC is particularly effective against BV-associated *Gardnerella* spp. biofilms, reducing their biomass and metabolism, at concentrations below 256 µg/mL. Scanning electron microscopy (SEM) revealed that DQC is also able to destroy the biofilm matrix architecture to some extent. Using fluorophore staining and confocal microscopy, we found that its mechanism of action does not seem to be associated with the primary lesion of the cytoplasmatic membrane.

DQC has already been described as an antimicrobial agent against *Gardnerella* spp. planktonic cells. In this study, we found that in addition to this antimicrobial activity, DQC is also active against *Gardnerella* spp. BV-associated biofilms, thus reducing their metabolic activity and to some extent their biomass. Since DQC has demonstrated good activity against planktonic cells of *Atopobium vaginae* [24] and *Candida albicans* [14], a similar effect on combined biofilms, like the ones formed on the vaginal mucosa, could be expected. However, scientific data to support this hypothesis are missing. On the other hand, the effect on *Gardnerella* spp. biofilms supports the beneficial properties of DQC in the treatment of BV infections, as the molecule is active against *Gardnerella* spp. biofilms and shows a low toxicity level. In contrast to clindamycin, DQC is not expected to induce the emergence of resistance in the microorganism cells.

## Figures and Tables

**Table 1:** Effective concentrations of dequalinium chloride in the destruction of bacterial vaginosis (BV) and non-BV associated *Gardnerella* spp. biofilms.

Strain	BV Status	Biomass EC_50_	Metabolic EC_50_
**085**	Non-BV	>256 µg/mL	81.01 µg/mL
**131**	Non-BV	>256 µg/mL	81.01 µg/mL
**246**	Non-BV	>256 µg/mL	81.01 µg/mL
**137**	BV	2.57 µg/mL	2.57 µg/mL
**241**	BV	8.11 µg/mL	8.11 µg/mL

## Data Availability

The data presented in this study are available on request from the corresponding author.

## Supplementary Materials

The following are available online at https://www.mdpi.com/2076-0817/10/3/261/s1, Figure S1: 3D representation, depicting the interaction of the test substance (dequalinium chloride, 256 μg/mL) with Gardnerella spp. biofilm of BV strain 241. Figure S2: Visualization of Gardnerella spp. biofilm of non-BV strain 131 and BV strain 241 after treatment with the test substance (dequalinium chloride, 256 μg/mL).

## References

[1] Machado D., Castro J., Palmeira-de-Oliveira A., Martinez-de-Oliveira J., Cerca N. (2015). Bacterial Vaginosis Biofilms: Challenges to Current Therapies and Emerging Solutions. Front. Microbiol..

[2] Dongari-Bagtzoglou A. (2008). Pathogenesis of mucosal biofilm infections: Challenges and progress. Expert Rev. Anti Infect.Ther..

[3] Pelling H., Nzakizwanayo J., Milo S., Denham E.L., MacFarlane W.M., Bock L.J., Sutton J.M., Jones B.V. (2019). Bacterial biofilm formation on indwelling urethral catheters. Lett. Appl. Microbiol..

[4] Miquel S., Lagrafeuille R., Souweine B., Forestier C. (2016). Anti-biofilm Activity as a Health Issue. Front. Microbiol..

[5] Castro J., Jefferson K.K., Cerca N. (2020). Genetic Heterogeneity and Taxonomic Diversity among *Gardnerella* Species. Trends Microbiol..

[6] Rosca A.S., Castro J., Sousa L.G.V., Cerca N. (2020). *Gardnerella* and vaginal health: The truth is out there. FEMS Microbiol. Rev..

[7] Muzny C.A., Taylor C.M., Swords W.E., Tamhane A., Chattopadhyay D., Cerca N., Schwebke J.R. (2019). An Updated Conceptual Model on the Pathogenesis of Bacterial Vaginosis. J. Infect. Dis..

[8] Castro J., Alves P., Sousa C., Cereija T., França A., Jefferson K.K., Cerca N. (2015). Using an in-vitro biofilm model to assess the virulence potential of bacterial vaginosis or non-bacterial vaginosis *Gardnerella vaginalis* isolates. Sci. Rep..

[9] Donders G.G., Zodzika J., Rezeberga D. (2014). Treatment of bacterial vaginosis: What we have and what we miss. Expert Opin. Pharmacother..

[10] Tomás M., Palmeira-de-Oliveira A., Simões S., Martinez-de-Oliveira J., Palmeira-de-Oliveira R. (2020). Bacterial vaginosis: Standard treatments and alternative strategies. Int. J. Pharm..

[11] Mayer B.T., Srinivasan S., Fiedler T.L., Marrazzo J.M., Fredricks D.N., Schiffer J.T. (2015). Rapid and Profound Shifts in the Vaginal Microbiota Following Antibiotic Treatment for Bacterial Vaginosis. J. Infect. Dis..

[12] McBain A.J., Ledder R.G., Moore L.E., Catrenich C.E., Gilbert P. (2004). Effects of Quaternary-Ammonium-Based Formulations on Bacterial Community Dynamics and Antimicrobial Susceptibility. Appl. Environ. Microbiol..

[13] Mendling W., Weissenbacher E.R., Gerber S., Prasauskas V., Grob P. (2016). Use of locally delivered dequalinium chloride in the treatment of vaginal infections: A review. Arch. Gynecol. Obstet..

[14] Della-Casa V., Noll H., Gonser S., Grob P., Graf F., Pohlig G. (2002). Antimicrobial activity of dequalinium chloride against leading germs of vaginal infections. Arzneim. Forsch..

[15] Babbs M., Collier H.O., Austin W.C., Potter M.D., Taylor E.P. (1956). Salts of decamethylene-bis-4-aminoquinaldinium (dequadin); a new antimicrobial agent. J. Pharm. Pharmacol..

[16] Weissenbacher E.R., Donders G., Unzeitig V., Tejada B.M., Gerber S., Halaška M., Špaček J. (2012). Fluomizin Study Group, A comparison of dequalinium chloride vaginal tablets (Fluomizin^®^) and clindamycin vaginal cream in the treatment of bacterial vaginosis: A single-blind, randomized clinical trial of efficacy and safety. Gynecol. Obstet. Investig..

[17] Castro J., Rosca A.S., Cools P., Vaneechoutte M., Cerca N. (2020). *Gardnerella vaginalis* enhances *Atopobium vaginae* viability in an in vitro model. Front Cell Infect Microbiol..

[18] Peeters E., Nelis H.J., Coenye T. (2008). Comparison of multiple methods for quantification of microbial biofilms grown in microtiter plates. J. Microbiol. Methods.

[19] Machado D., Palmeira-de-Oliveira A., Cerca N. (2015). Optimization of culture conditions for *Gardnerella vaginalis* biofilm formation. J. Microbiol. Methods.

[20] Wilson D.N. (2013). Ribosome-targeting antibiotics and mechanisms of bacterial resistance. Nat. Rev. Microbiol..

[21] Austin M.N., Beigi R.H., Meyn L.A., Hillier S.L. (2005). Microbiologic response to treatment of bacterial vaginosis with topical clindamycin or metronidazole. J. Clin. Microbiol..

[22] Hugo W.B., Frier M. (1969). Mode of action of the antibacterial compound dequalinium acetate. Appl. Microbiol..

[23] Belosludtsev K.N., Belosludtseva N.V., Tenkov K.S., Sharapov V.A., Kosareva E.A., Dubinin M.V. (2018). Effect of Dequalinium on Respiration and the Inner Membrane Permeability of Rat Liver Mitochondria. Biochem. (Mosc.) Suppl. Ser. A Membr. Cell Biol..

[24] Santiago G.L.S., Grob P., Verstraelen H., Waser F., Vaneechoutte M. (2012). Susceptibility testing of *Atopobium vaginae* for dequalinium chloride. BMC Res. Notes.

