# Supplementary material for: Dequalinium Chloride Effectively Disrupts Bacterial Vaginosis (BV) Gardnerella spp. Biofilms
Source: Pathogens. 2021 Feb 25;10(3):261. doi: 10.3390/pathogens10030261 (PMC7996269; doi:10.3390/pathogens10030261)
Supplement: Supplementary file 1 [file pathogens-10-00261-s001.pdf]

pathogens

MDPI

Supplemental Material

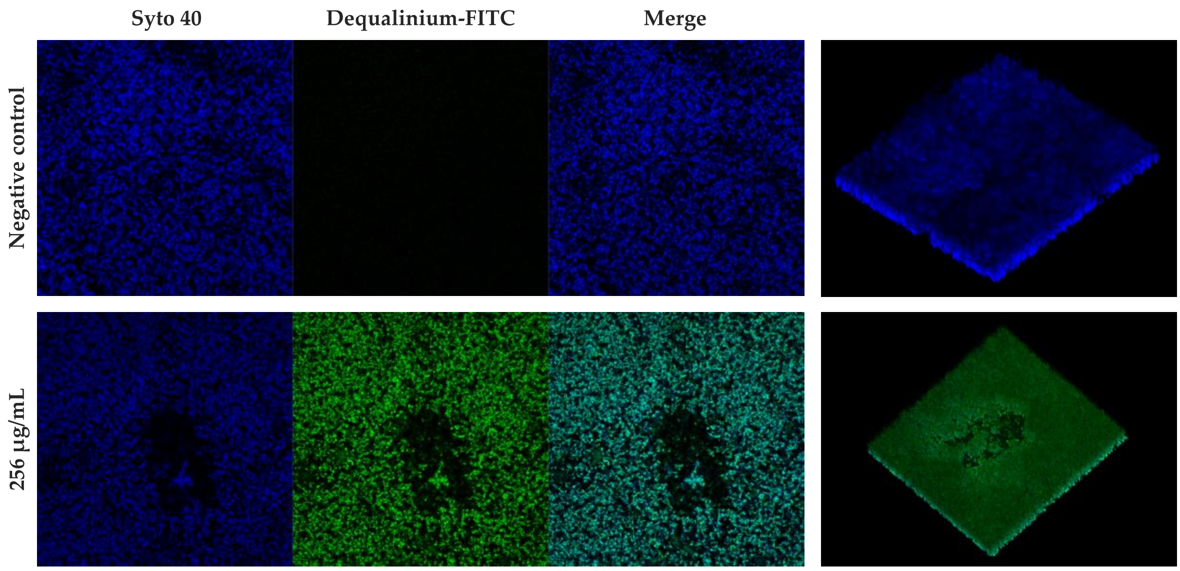

Figure S1. 3D representation, depicting the interaction of the test substance (dequalinium chloride, 256 µg/mL) with *Gardnerella* spp. biofilm of BV strain 241. Green: dequalinium chloride (FITC), Blue: nucleic acids (Syto40); Red: dead cells (propidium iodide, PI). Live: positive control, culture media was used to form biofilms. Dead: 70% ethanol was used to kill formed biofilms. Dequalinium chloride: treated biofilms with the dequalinium chloride-FITC complex.

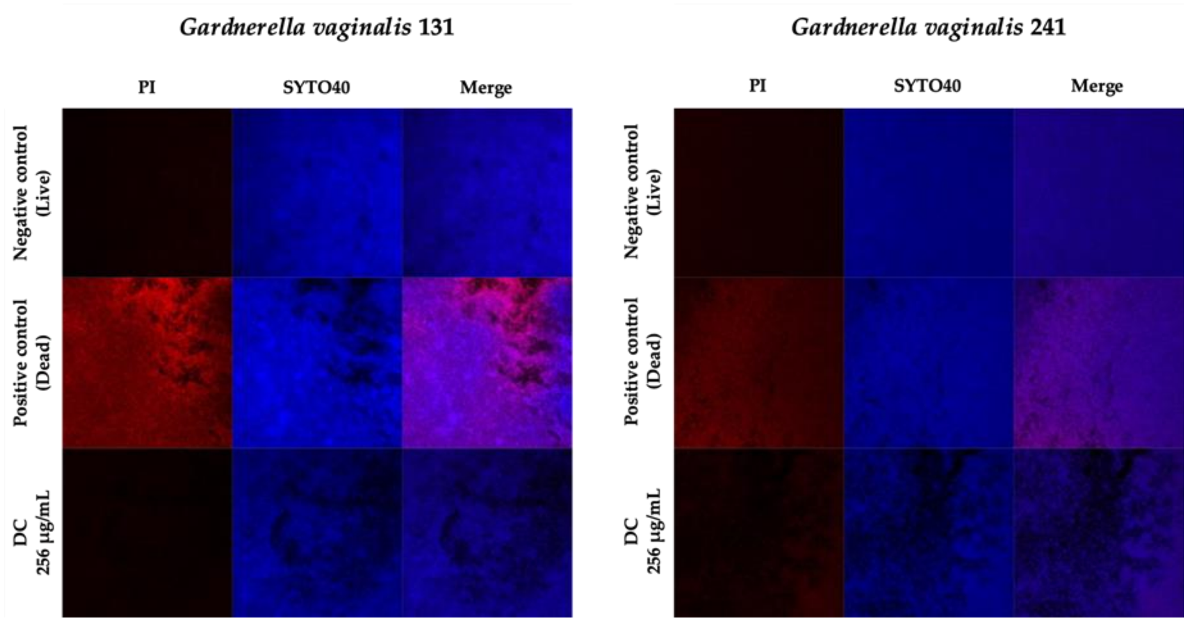

Figure S2. Visualization of *Gardnerella* spp. biofilm of non-BV strain 131 and BV strain 241 after treatment with the test substance (dequalinium chloride, 256 µg/mL). Blue: nucleic acids (Syto40); Red: dead cells (propidium iodide, PI). Live: negative control, culture media was used to form biofilms. Dead: positive control, 70% ethanol was used to kill formed biofilms. DC: treated biofilms with dequalinium chloride.
